# Methods and challenges in measuring the impact of national pneumococcal and rotavirus vaccine introduction on morbidity and mortality in Malawi

**DOI:** 10.1016/j.vaccine.2015.04.053

**Published:** 2015-05-28

**Authors:** Naor Bar-Zeev, Lester Kapanda, Carina King, James Beard, Tambosi Phiri, Hazzie Mvula, Amelia C. Crampin, Charles Mwansambo, Anthony Costello, Umesh Parashar, Jacqueline E. Tate, Jennifer R. Verani, Cynthia G. Whitney, Robert S. Heyderman, Nigel A. Cunliffe, Neil French, for the VacSurv Consortium

**Affiliations:** a Malawi-Liverpool-Wellcome Trust Clinical Research Programme, College of Medicine, University of Malawi, Blantyre, Malawi; b Institute of Infection & Global Health, University of Liverpool, Liverpool, UK; c Mai Mwana Project, Mchinji, Malawi; d Institute for Global Health, University College London, London, UK; e Karonga Prevention Study, Chilumba, Malawi; f London School of Hygiene & Tropical Medicine, London, UK; g Ministry of Health, Government of Malawi, Lilongwe, Malawi; h Centers for Disease Control and Prevention, Atlanta, GA, USA; i Liverpool School of Tropical Medicine, Liverpool, UK

**Keywords:** ARIa, cute respiratory infection, DSSd, emographic surveillance system, EPIe, xpanded program on immunization, GAVIg, lobal alliance for vaccines and immunization, IMCIi, ntegrated management of childhood illness, IPD, invasive pneumococcal disease, MEOm, onitoring & evaluation officer, NITAGn, ational immunisation technical advisory group, PCVp, neumococcal conjugate vaccines, PCV13, 13-valent pneumococcal conjugate vaccine, QECH, Queen Elizabeth Central Hospital, RV1m, onovalent rotavirus vaccine, WHO, World Health Organization, Vaccine, Effectiveness, Impact, Pneumococcal, Rotavirus, Malawi

## Abstract

**Background:**

Pneumonia and gastroenteritis are leading causes of vaccine-preventable childhood morbidity and mortality. Malawi introduced pneumococcal conjugate and rotavirus vaccines to the immunisation programme in 2011 and 2012, respectively. Evaluating their effectiveness is vital to ensure optimal implementation and justify sustained investment.

**Methods/Design:**

A national evaluation platform was established to determine vaccine effectiveness and impact in Malawi. Impact and effectiveness against vaccine-type invasive pneumococcal disease, radiological pneumonia and rotavirus gastroenteritis are investigated using before-after incidence comparisons and case-control designs, respectively. Mortality is assessed using a prospective population cohort. Cost-effectiveness evaluation is nested within the case-control studies. We describe platform characteristics including strengths and weaknesses for conducting vaccine evaluations.

**Discussion:**

Integrating data from individual level and ecological methods across multiple sites provides comprehensive information for policymakers on programme impact and vaccine effectiveness including changes in serotype/genotype distribution over time. Challenges to robust vaccine evaluation in real-world conditions include: vaccination ascertainment; pre-existing rapid decline in mortality and pneumococcal disease in the context of non-vaccine interventions; and the maintenance of completeness and quality of reporting at scale and over time. In observational non-randomised designs ascertainment of vaccine status may be biased particularly in infants with fatal outcomes. In the context of multiple population level interventions targeting study endpoints attribution of reduced incidence to vaccine impact may be flawed. Providing evidence from several independent but complementary studies will provide the greatest confidence in assigning impact. Welcome declines in disease incidence and in child mortality make accrual of required sample sizes difficult, necessitating large studies to detect the relatively small but potentially significant contribution of vaccines to mortality prevention. Careful evaluation of vaccine effectiveness and impact in such settings is critical to sustaining support for vaccine programmes. Our evaluation platform covers a large population with a high prevalence of HIV and malnutrition and its findings will be relevant to other settings in sub-Saharan Africa.

## Highlights

• Evaluation of vaccine impact and effectiveness is critically important but methodologically challenging.
• Challenges include achieving unbiased ascertainment of vaccine status, and non-vaccine-attributable decline in morbidity and mortality.
• Use of multiple sites together with diverse evaluation methods provides more robust evaluation.

## 1 Background

Key to reducing child mortality in developing countries has been the recent introduction of pneumococcal conjugate and rotavirus vaccines [1]. Their incorporation into routine infant schedules requires robust evidence from randomised efficacy trials. Demonstrating subsequent effectiveness and impact is challenging but essential for optimal implementation of these vaccines and sustained investment [2].

Pneumococcus is a leading cause of pneumonia, meningitis and bacteraemia [3]. In clinical trials pneumococcal conjugate vaccines (PCV) were extremely efficacious at preventing serious vaccine serotype disease [4–6]. In low disease burden high income countries, PCV have led to major public health benefits through direct effects and through herd immunity [7]. However, given differing epidemiology of pneumococcal transmission and exposure and vaccine scheduling in resource-poor settings and higher prevalence of malnutrition and HIV, vaccine effectiveness may differ. Furthermore, in several populations the overall benefit of PCV has been offset by an increase in non-vaccine serotype disease and doubts remain over the efficacy of the vaccine against some serotypes [8,9].

Rotavirus is the principal cause of severe diarrhoea globally and a major contributor to diarrhoeal deaths in the developing world [10]. Trials of oral rotavirus vaccines in high and middle income countries demonstrated high efficacy (>85%), with evidence of substantial public health impact demonstrated by post-introduction surveillance studies [11]. In low income countries these vaccines show lower efficacy [12,13]. A randomised trial of rotavirus vaccine conducted in Malawi found 49% efficacy [12], lower than in other studies [14,15]. Nonetheless, given the much higher disease burden and mortality, rotavirus vaccination has the potential to contribute to greater absolute improvements in child survival, particularly if indirect effects are apparent.

PCV and rotavirus vaccine were recommended by the World Health Organisation (WHO) in 2007 and 2009 respectively and subsequently became eligible for Gavi Alliance support for low income countries [16,17]. Malawi is among the poorest and most densely populated countries in Africa with a high burden of undernutrition, HIV, malaria, pneumonia and diarrhoea [18]. Increasing HIV prevalence among women age 15–49 of 13.3% in 2004 [19] to 22.7% in 2010 [20] led Malawi to adopt Option B+ (whereby all HIV-positive pregnant or breastfeeding women commence lifelong full antiretroviral therapy regardless of clinical or immunological stage) in 2011 [21,22]. Under 5 mortality is decreasing (79 per thousand live births in 2010) possibly due to increased birth spacing, antiretroviral roll-out, improved nutrition, sanitation and case management, placing Malawi on track to meet the fourth Millennium Development Goal of reducing child mortality [20]. With GAVI support, 13-valent pneumococcal conjugate vaccine (PCV13) was introduced into the national immunisation programme in November 2011 as a three-dose schedule (6, 10 and 14 weeks) with a catch-up dose in the first year of introduction for all infants <12 months old. Monovalent rotavirus vaccine (RV1) was introduced in October 2012 on a WHO recommended two-dose schedule (6 and 10 weeks).

Surveillance for invasive pneumococcal disease (IPD) or rotavirus acute gastroenteritis exists elsewhere in Africa, including the Gambia, South Africa, Ghana and Kenya amongst others [23,24]. Malawi is notable for being the only country in which PCV13 was introduced to a pneumococcal vaccine naïve population without a catch-up campaign after the first year of life, and for investigating the impact of both PCV and RV1 on infant mortality. We describe the methods of a nationwide surveillance platform that has been established to evaluate pneumococcal and rotavirus vaccine impact and effectiveness and consider the platform's strengths and weaknesses.

## 2 Methods

### 2.1 Study design

In collaboration with the Ministerial National Immunisation Technical Advisory Group (NITAG) [25], a national vaccine evaluation programme for vaccine-preventable endpoints has been established in Malawi to determine the population impact of new vaccines on mortality and morbidity. Several interlinked epidemiological methods have been used (Table 1):

1. Sentinel surveillance at one rural and one urban site to investigate changing disease incidence and distributions of *S. pneumoniae* serotypes and rotavirus genotypes.
2. Matched case-control studies of direct PCV effectiveness against IPD and against radiological pneumonia among vaccine age-eligible children using age-neighbourhood matched community controls selected by random walk.
3. Matched case-control study of indirect PCV effectiveness against IPD in adults living with vaccine age-eligible children compared with community based adult controls also living with vaccine eligible children.
4. Matched and unmatched case-control studies of RV1 effectiveness against severe acute rotavirus gastroenteritis using age-neighbourhood matched community controls and unmatched hospitalised rotavirus test-negative controls respectively [26–28].
5. Population based cohort study to measure <5 years and post-neonatal infant all-cause and disease-specific mortality by vaccine status [29].
6. Cost-effectiveness studies from a societal, health care provider and household perspective collecting comprehensive itemised costing of actual costs incurred [30].

Case definitions, screening criteria and investigations are shown in Tables 2 and 3. Universally issued subject- held medical records (health passports) which contain the vaccination records are interrogated for vaccination status.

### 2.2 Sites and populations under surveillance

**Fig. 1:**
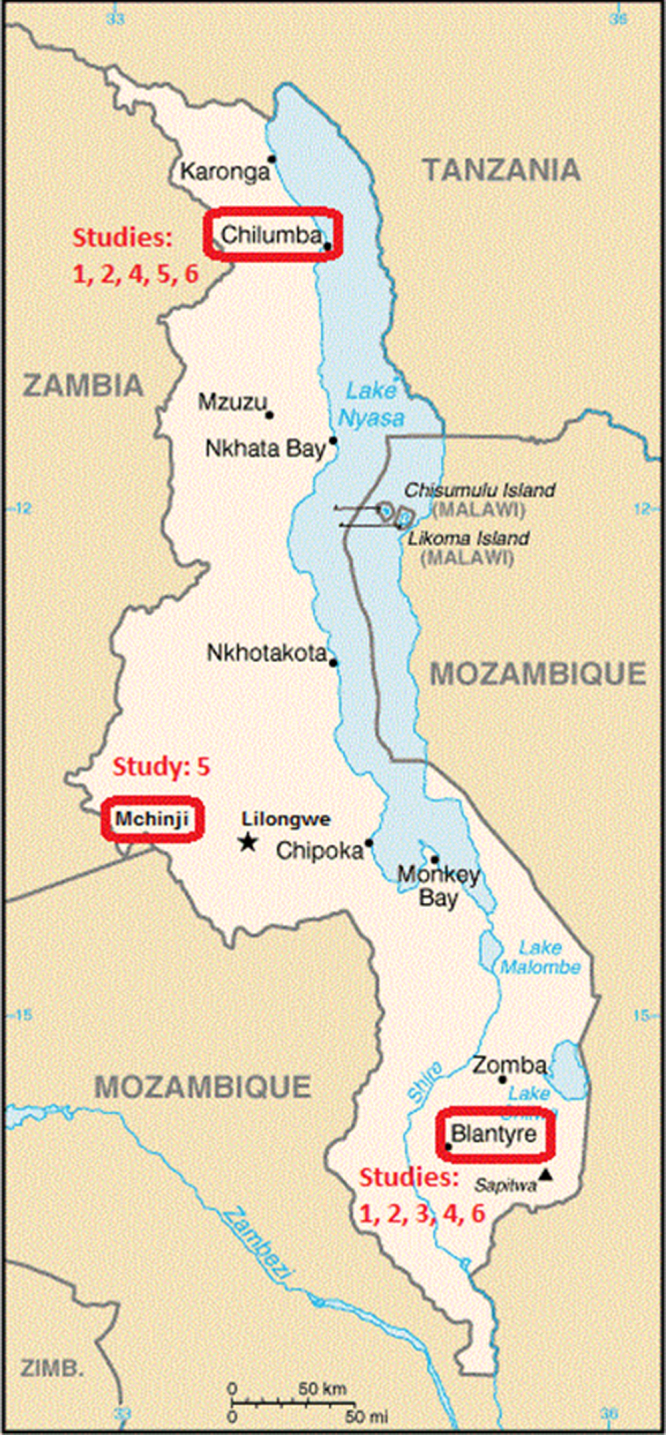
Map of Malawi. Areas highlighted represent the three study sites. Studies by site: (1) Disease incidence and distributions of *S. Pneumonia* serotypes and rotavirus genotypes. (2) Matched case-control studies of direct PCV effectiveness against IPD and against radiological pneumonia using community controls. (3) Matched case-control study of indirect PCV effectiveness against IPD in adults living with vaccine age-eligible children. (4) Matched and unmatched case-control studies of RV1 effectiveness against severe acute rotavirus gastroenteritis using community controls and hospitalised rotavirus test-negative controls, respectively. (5) Population based cohort study to measure under-5 and post-neonatal infant all-cause and disease-specific mortality by vaccine status. (6) Cost-effectiveness studies from societal, healthcare provider and household perspectives.

The northern, central and southern regions of Malawi all participate in surveillance; the northern (Karonga) and central (Mchinji) sites are rural locations. The southern site (Blantyre) is a large city with high-density urban populations and a peri-urban rural penumbra, so surveillance is representative of the entire population and demography of Malawi (Fig. 1). The mortality cohort study is located in the northern and central regions, while the morbidity case control studies and cost-effectiveness studies are located in the northern and southern regions.

*Northern Malawi:* Supported by the London School of Hygiene & Tropical Medicine, the Karonga Prevention Study demographic surveillance system covering 35,000 individuals has operated in Karonga district since 2002 [31], health facility based pneumonia and diarrhoea surveillance in children operating since 2008 [31]. Births and deaths are reported monthly by a system of village key informants with verbal autopsies performed on all deaths. Health passport vaccine records are reviewed at an annual census. Children under surveillance attend Chilumba Rural Hospital for medical care. Those with Integrated Management of Childhood Illness (IMCI) defined severe pneumonia or gastroenteritis undergo chest X-ray or stool collection for rotavirus testing, blood cultures are not performed. Karonga contributes to studies 1, 2 (but not IPD), 4 and 5 listed above. The study site provides robust data on historical rates of disease and mortality prior to vaccine introduction.

**Fig. 2:**
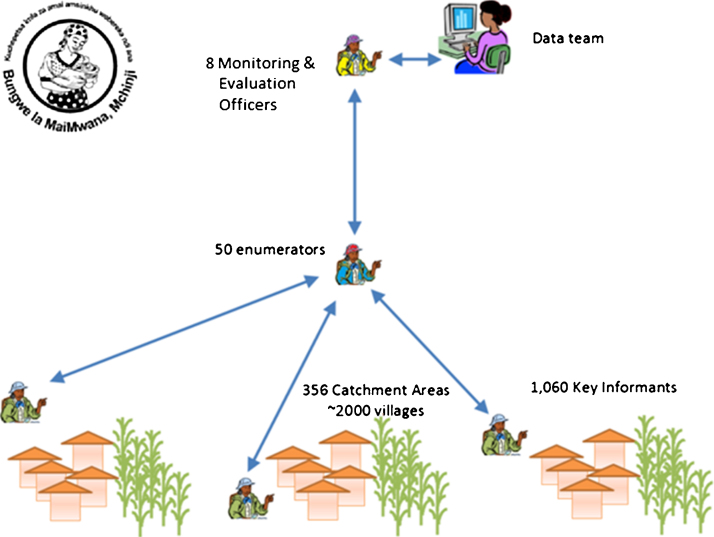
Structure of data flow for the Mchinji Mortality Cohort Study.

*Central Malawi:* Mchinji district population was 456,516 persons in the 2008 census, with an estimated live birth rate of 57 per 1000 population [32,33]. We conducted a baseline census of Mchinji district in March 2012. Ongoing prospective surveillance of over 2000 villages in the district is conducted for births and deaths or migration of children under 5 and women of child-bearing age. Village informants report vital events monthly, supported by enumerators, supervised by monitoring and evaluation officers (Fig. 2). Live births are followed to 1 year of age or death or emigration from the district. All deaths reported by informants are verified and cause of death determined by verbal autopsy. Vaccine status is obtained from the health passport at household visits at 4 and 12 month of age or following death. Mchinji contributes to the population based cohort study listed above (study 5) and the primary endpoint of survival to 1 year. The site is coordinated by the Mai Mwana Project, with support from University College London.

*Southern Malawi:* The Malawi-Liverpool-Wellcome Trust Clinical Research Programme (MLW) in Blantyre has surveillance for IPD spanning 13 years [3,22], and has extensively characterised rotavirus molecular epidemiology in the pre-vaccine era [34,35]. Blantyre district numbered 1,001,984 persons at the 2008 census [32]. Queen Elizabeth Central Hospital (QECH) provides free health services and is the principal referral centre [36]. Active case finding at QECH paediatric emergency department and inpatient wards enrols children with presumed sepsis, meningitis, acute gastroenteritis and severe respiratory illness, in whom blood culture, lumbar puncture, stool collection, chest radiography or nasopharyngeal swabs are performed per protocol. Blood culture is not routinely performed in pneumonia unless a clinical suspicion of sepsis exists. Anthropometry, vaccination status, and HIV status are recorded [21]. This site is the platform for case-control studies of PCV13 effectiveness against vaccine type IPD and against radiological pneumonia and of RV1 against severe rotavirus gastroenteritis. The Blantyre site contributes to studies 1, 2, 3, and 4 listed above.

### 2.3 Sample size estimates

*Vaccine effectiveness case-control studies:* Assuming 60% vaccination coverage and control:case ratio of 4, the studies have 80% power to detect PCV13 effectiveness of ≥60% against vaccine type IPD with 47 vaccine age-eligible cases, ≥30% effectiveness against radiologically confirmed pneumonia with 311 cases, and effectiveness of ≥40% RV1 effectiveness against severe acute rotavirus gastroenteritis with 82 cases. Among adults, 311 vaccine type IPD cases living with vaccine eligible children are needed to detect ≥30% indirect effectiveness.

*Mortality cohort study:* Assuming 70% vaccination coverage and accounting for a 12% loss to follow-up before 1 year of age, we aim to recruit 38,213 live births over 2 years. This cohort provides 80% power at 5% one-tailed significance to detect vaccine associated improvement in cause-specific post-neonatal infant mortality of ≥36% if baseline mortality is 15 per 1000 live births.

*Cost-effectiveness*: A sample size of 88 for each clinical endpoint (confirmed rotavirus gastroenteritis, radiologically confirmed pneumonia) provides a precise estimator of the mean cost of illness episode to within a margin ±10% [37].

### 2.4 Implementation

Initial programme design involved close collaboration with the Ministry of Health, with specific inputs from the following offices: Expanded Programme for Immunisation, Acute Respiratory Infection branch, IMCI branch and Epidemiology branch. Site visits were conducted to assess research capacity. Regular meetings were held with the District Health Offices and Traditional Authorities. Study oversight is provided by the national coordinator based at MLW Blantyre, with site coordinators at each location. Regular site visits occur.

Study instruments were translated to Chichewa and Tumbuka. Piloting took one month in Karonga, nine in Mchinji and two in Blantyre. Surveillance for rotavirus gastroenteritis commenced in August 2011 in Karonga and January 2012 in Blantyre, and for child mortality in Mchinji in March 2012. Case control studies for IPD and rotavirus commenced on the dates of vaccine introduction, but for radiological pneumonia commencement was in September 2013, approximately 23 months after vaccine introduction.

### 2.5 Capacity building and health system integration

All studies have dedicated employed staff. Clinical staff were trained in IMCI clinical management, Good Clinical Practice and formally qualified as HIV testers and counsellors. Training in paediatric radiological pneumonia standards was done against a WHO defined panel of standard films [38]. WHO verbal autopsy training was undertaken in Mchinji and Karonga. Sensitisation on childhood immunisation of community leaders and health care staff was undertaken. Review and feedback on quality of recording in vaccination centre registers was also conducted. Data are shared with the NITAG in which senior study staff are members, and Ministerial NITAG members are on the platform's steering committee.

### 2.6 Physical resources

*Field:* Coordination of activities across distant sites with poor accessibility demanded a robust motorbike and bicycle based transport infrastructure and regular site visits by supervisors to local field teams for scheduled data monitoring visits.

*Laboratory:* MLW has an external quality assured microbiology laboratory. Stool specimens received in Karonga are transported to MLW for further processing. Rotavirus status is determined by enzyme linked immunosorbent assay (Rotaclone™, Meridian Bioscience, Cincinnati). Rotavirus genotyping is done using hemi-nested multiplex RT-PCR [39]. Pneumococcal quantification is performed using *lytA* PCR, while serotyping of carriage specimens from Karonga and Blantyre and of invasive isolates from Blantyre is performed using triplex PCR and latex agglutination [40]. HIV status of all children participating in studies 1, 2, 3, 4 and 6 is determined in accordance with national guidelines [21].

*Radiology*: Chest X-rays from Blantyre and Karonga are stored digitally according to a standardised protocol. Digital files are reviewed at each site and then centrally by a trained panel.

### 2.7 Data management

Data management has ensured harmonised linkage among the sites, whilst integrating with existing legacy systems [41]. Data are captured on paper forms and entered to a relational Structured Query Language database, with embedded data checks. In Mchinji paper forms are reviewed, scanned and entered. Timeliness is critical since subsequent contacts with subjects are automatically triggered by the database. Verbal autopsy data are captured using purpose built Visual Basic (Microsoft) platform. Similar systems occur in Karonga. Data cleaning, merging, logging and archiving is undertaken weekly by a dedicated team, whose performance is monitored. Subject recruitment by endpoint is reported at each site and is reviewed regularly by the study steering committee.

### 2.8 Quality assurance activities

For valid ascertainment of vaccine status, the health passport is reviewed upon each contact with participants. Passport images are scanned and digitally archived. In Karonga, digital capture occurs for all cases and a random selection of children in the DSS. In Mchinji, cluster sampling by catchment area is undertaken and a representative sample is imaged and archived. Vaccination records are checked against the local health centre vaccination register. Parental verbal report is also obtained for all children. Discrepancies are resolved on the basis of rules defined a priori in the study protocol. In case of missing passports, particularly among deceased infants, vaccination registers are cross checked. In Mchinji, interviews are conducted quarterly with a sub-set of mothers following vaccination visits to confirm whether the child received the number of vaccine doses that are recorded in the health passport.

### 2.9 Programme performance indicators

Recruitment and protocol compliance are reviewed weekly in Blantyre, and monthly in Karonga and Mchinji. Observed event rates are compared to long term expected rates and possible under-ascertainment is investigated by detailed review. Contiguous periods of suspected under-reporting will trigger the central database to issue a warning and restorative field activities undertaken to collect missing data.

### 2.10 External evaluation

Initial study protocols were reviewed by an external scientific panel on behalf of the Wellcome Trust. Subsequent formal regular external review of protocol compliance is provided by visiting epidemiologists (separate from study co-investigators) from the Centers for Disease Control and Prevention, Atlanta.

### 2.11 Ethics

Community approvals from Traditional Authorities and Community Health Management Teams were obtained prior to study implementation and regular feedback of study progress is given. Informed written consent is obtained from parents for case-control studies. Verbal consent is obtained for vital events surveillance and verbal autopsy. Ethical approval was provided by the Malawian National Health Sciences Research Committee and University of Malawi Research Ethics Committee, and by the University of Liverpool and London School of Hygiene & Tropical Medicine.

## 3 Discussion

Maintaining high quality real world surveillance at large scale is challenging [42]. We outline the strengths and weaknesses of our vaccine evaluation platform.

### 3.1 Strengths

Incorporating established large-scale population based surveillance, the program covers a very large population, spanning urban, peri-urban and rural settings. It has the capacity to determine vaccine effectiveness against mortality and morbidity in the context of high rates of malnutrition and HIV. Because the studies include rural and urban populations and span primary to referral level care, the results are likely to be relevant to other countries in sub-Saharan Africa or to low income countries elsewhere. Study sites have strong community engagement and acceptance, and have high quality track records in population epidemiology and clinical trials. Strong laboratory surveillance capacity has been well established at MLW for over a decade, allowing pneumococcal serotype and rotavirus genotype distributions to be described before and after respective vaccine introductions [3,22].

In contrast to vaccine evaluations elsewhere, studies in Malawi are occurring in a pneumococcal and rotavirus vaccine naïve population, so can determine changes in pneumococcal serotype and rotavirus genotype distribution for currently available vaccines. Using multiple sites and methods provides complementary and independent measures of vaccine effectiveness. Measures of effectiveness against specific (e.g. IPD, RV proven gastroenteritis) and less specific endpoints (e.g. radiological pneumonia, all-cause mortality) are of major public health importance, and address questions of value to policy makers. Nesting of multiple observational studies examining different endpoints and spanning several sites provides more robust evidence of impact than any single study could provide. Involvement of multiple agencies within the Ministry of Health and its NITAG ensures integration of studies within existing government programmes thus engendering strong community engagement and acceptability.

### 3.2 Challenges

Vaccine ascertainment is a major challenge (Table 4). Measurement of vaccination status depends predominantly on health passports, the availability of which may be biased [43]. We use multiple methods to obtain recorded documentation of vaccination including home visits and cross-checking against health centre vaccination registers. Retention of health passports among living vaccine age-eligible children at 1 year of age is high (93% in Mchinji and 85% in Karonga) [43]. There is however, a common practice in Malawi to bury the health passport with the deceased child. We obtain vaccination status as early as possible following schedule completion and cross check against clinic registers in an attempt to mitigate this problem.

Another challenge is determining vaccine effect in the setting of multiple interventions. Malawi has seen a decline in pneumococcal disease and in infant and child mortality over the past decade [3,20,44]. Attributing any observed reduction in morbidity or mortality to vaccine introduction, compared with the historical trend is difficult. Multiple government and non-government funded interventions have been implemented in Malawi. In Karonga for example, large scale sanitation efforts were implemented around the introduction of rotavirus vaccine. Such interventions, their scope and coverage affect the assessment of vaccine programme impact, and are difficult to adjust for in analysis. The study methods primarily determine concurrent individual level assessment of vaccine effectiveness by vaccine receipt, adjusting for individual and household exposure to communal interventions. Before and after analysis is also informative but attribution of any observed changes to vaccine alone is problematic.

Dealing adequately with confounders that increase the risk of disease outcomes among unvaccinated children independent of vaccine receipt is difficult. As population coverage rises the unvaccinated group becomes less representative of the general population. We measure socioeconomic and demographic covariates and adjust for these, but cannot deal with unmeasured confounders in the absence of randomisation. For incidence analysis we will conduct propensity scoring to help account for ascertainment bias arising from differential care-seeking behaviours, and adjust for prevalence of HIV, malnutrition and malaria [3,22,45].

Obtaining adequate sample size is a problem for some studies. The decline in IPD makes achieving recruitment targets in case-control designs difficult. This has led to the abandonment of the case-control design to evaluate indirect effects (Study 3) and increases the importance of ecological impact evaluation, but with the latter design attribution of any incidence reduction to vaccine must be cautious. Given our observed baseline mortality was lower than was originally anticipated based on published estimates [20], we will only be powered for high end decreases in all-cause mortality of greater magnitude than previously observed [6], increasing the importance of having independent measures to provide consistency. In addition, the introductions of PCV13 and RV1, were separated by 11 months. Reductions in all-cause mortality achieved by PCV13 will make detecting a benefit of RV1 more difficult, so cause-specific mortality must be used which introduces a level of uncertainty regarding the precision of causal determination by verbal autopsy. Although we will examine the impact of HIV on vaccine effectiveness, we are not adequately powered for definitive conclusions regarding this important issue.

Completeness and quality of reporting is a continual challenge. With over 1000 field workers, heterogeneity in data quality is inevitable. Supportive supervision, simple data collection instruments and automated performance target triggers are used to improve data reporting by key informants and rectify under-performing catchment areas. Long term sustainability of funding, which is currently borne through competitive research funding mechanisms, remains a challenge [46].

### 3.3 Interpretation

Maintaining large scale integrated multi-site epidemiological research of high quality is challenging, requiring ongoing investment in quality assurance of data collection. Reliance on existing data systems like health passports and vaccine registers is hampered by incomplete recording and by cultural factors, so measuring vaccine status requires early proactive effort if observation bias is to be minimised. Formally imputing vaccine status to deceased children is also subject to biases arising from our imputation modelling assumptions. Community engagement within existing socio-cultural systems is critical to the acceptability of surveillance. Using multimodal methods of analysis that include individual evaluation of effectiveness and ecological evaluation of impact is important, particularly for the detection of indirect vaccine effect. Integrating several strands of evidence with consistent results will provide greater confidence in surveillance findings for policy makers to act on. Other countries should of course consider their own epidemiologic settings when evaluating the generalisability of our results.

A long-term challenge facing this work and identified by the Global Framework for Immunization Monitoring and Surveillance is financial sustainability [46]. Establishing the early public health benefits following vaccine introduction produces data to advocate for broader vaccine use. Long term monitoring, though more prosaic, is no less important since it maintains the quality of the vaccination programme. By building this surveillance system collaboratively with the Malawi national immunization programme, utilising established data sources, training local health staff, and establishing locally feasible data management systems we have gone some way to meet the Global Framework requirements, but sources of continued funding will be required.

## 4 Conclusion

Despite the challenges, evaluating how effective new vaccines are in a real-world context is critical to encouraging vaccine introductions and sustaining vaccination programmes. Our large-scale surveillance system provides a platform for evaluating impact of PCV13 and RV1 on infant mortality and morbidity from IPD, radiologically confirmed pneumonia and severe gastroenteritis, and will determine cost-effectiveness in one of the world's poorest countries. The system is powerful and of high quality but faces considerable challenges, reflecting the real-world difficulties of conducting high quality large scale research in infrastructure-poor settings. Demonstrating an impact on mortality (due 2016) and morbidity (due 2015) will be important for the long term investment required to sustain vaccination programmes and to maximise their reach and delivery.

## Competing interests

NF, NBZ and NAC obtained an investigator-led project grant from Glaxo-Smith-Kline Biologicals.

## Authors’ contributions

NBZ, NAC, RSH & NF conceived this paper. NBZ, LK, CK, JB, HM acquired the data and were responsible for data management. TP, ACC, CM, AC, UP, JET, JRV, CGW assisted in analysis and interpretation. NBZ wrote the first draft. All authors read, critically revised and approved the final manuscript.

## Figures and Tables

**Table 1:** Summary of study details.

Study	Design	Primary endpoint (see also Table 3)	Sample size	Study site	Recruitment to date	Anticipated completion
1	Sentinel surveillance	Pneumococcus: • Serotype specific incidence of invasive pneumococcal disease • Relative serotype abundance in carriage • Clinical and radiological pneumonia Rotavirus: Genotypic specific incidence of clinical gastroenteritis	Not applicable	Blantyre (IPD) Blantyre & Karonga (carriage, pneumonia, rotavirus)	Not applicable	Ongoing
2a	Matched case-control studies of direct PCV effectiveness against IPD	Odds ratio of vaccine receipt among cases and matched controls	47 VT IPD	Blantyre	20 IPD	Uncertain
2b	Matched case-control studies of direct PCV effectiveness against radiological pneumonia	Odds ratio of vaccine receipt among cases and matched controls	311 RCP	Blantyre & Karonga	855 clinical pneumonia, 180 RCP	2016
3	Matched case-control study of indirect PCV effectiveness against IPD in adults living with vaccine age-eligible children compared with community based adult controls also living with vaccine eligible children	Odds ratio of vaccine receipt among age-eligible infants living in household of adult cases and controls	311	Blantyre		Abandoned
4	Matched and unmatched case-control studies of RV1 effectiveness against severe acute rotavirus gastroenteritis using community controls and hospitalised rotavirus test-negative controls respectively	Odds ratio of vaccine receipt among cases and matched community controls Odds ratio of vaccine receipt among cases and test-negative diarrheic controls	102	Blantyre & Karonga	109	Completed [28]
5	Population based cohort study to measure <5 years and post-neonatal infant all-cause and disease-specific mortality by vaccine status	Hazard ratio of all-cause and cause-specific post-neonatal infant mortality, by vaccine receipt.	38,213	Mchinji & Karonga	34,119 PCV13 eligible 25,238 RV1 eligible	2016
6	Cost-effectiveness studies	Cost per disability adjusted life year saved	88 for each vaccine	Blantyre & Karonga	530	Completed

VT = vaccine type, IPD = invasive pneumococcal disease, RCP = radiographically confirmed pneumonia, PCV13 = 13-valent pneumococcal conjugate vaccine, RV1 = monovalent rotavirus vaccine.

**Table 2:** Screening criteria for clinical syndromes.

Clinical syndrome	Screening criteria	Investigation performed
Gastroenteritis	History of 3 looser than usual stools in 24 h	Stool collection for rotavirus EIA
Pneumonia (IMCI-defined)	Raised respiratory rate for age AND any of: history of cough or difficulty breathing	Nasopharyngeal swab with serotyping of identified pneumococci Chest X-ray
Severe pneumonia	**Pneumonia** AND any of: chest wall indrawing or nasal flaring or grunting	Nasopharyngeal swab Chest X-ray
Hypoxaemic pneumonia	**Pneumonia** AND oxygen saturation <90% in air	Nasopharyngeal swab Chest X-ray
Very severe disease (IMCI-defined)	Any of: History of convulsion, *or* Impaired consciousness (on Blantyre Coma Score[40]), *or* Inability to feed, *or* Vomiting everything, *or* Weight for age ≤ −3 standard deviation from mean	Blood culture, with serotyping* of identified pneumococci If suspected meningitis: Lumbar puncture with biochemistry, cell count, Gram stain and culture. Purulent culture negative cases have *lytA* PCR. If associated pneumonia: Nasopharyngeal swab Chest X-ray
**Suspected sepsis**	Axillary temperature of at least 38 °C or less than 36 °C AND **Very severe disease;***or* Any child with clinical suspicion of sepsis.	Blood culture, with serotyping* of identified pneumococci Malaria parasites screen
**Suspected meningitis**	Bulging fontanelle, *or* Stiff neck, *or* **Very severe disease** with clinically suspicion of meningitis, *or* **Suspected sepsis** with clinical suspicion of meningitis	Lumbar puncture Blood culture, with serotyping* of identified pneumococci

* Serotyping by real time PCR and latex agglutination.

**Table 3:** Primary endpoints for 4 vaccine effectiveness studies.

Primary endpoint	Case definition
**13-valent vaccine-serotype invasive pneumococcal disease**	A suspected episode of septicaemia or meningitis with confirmed aetiology from CSF or blood by isolation of pathogen or detection by PCR based methods.
**WHO defined radiological pneumonia**	Among children <5 years presenting to a study site (either as outpatient or admitted inpatient), an episode of IMCI-defined (see Table 1) severe pneumonia or pneumonia with very severe disease that also has WHO defined endpoint consolidation as determined by concordant X-ray reading among a panel of 3 especially trained readers.
**Severe acute rotavirus gastroenteritis**	New onset of gastroenteritis with a modified Vesikari score ≥11 and with laboratory confirmation of rotavirus infection by enzyme linked immunosorbent assay.
**All-cause post-neonatal infant mortality**	A reported death of a child aged 4 to 52 weeks from the defined birth cohort capture population.

**Table 4:** Challenges to valid vaccine evaluation at scale in Malawi, their potential impact and strategies to mitigate these.

Challenge	Potential impact	Mitigation strategies
Differential vaccine status ascertainment	• Reduce power if rely solely on written record. • Potential bias if rely also on parental recall	• Early ascertainment by study staff • Community sensitisation on importance of record retention • Cross checking against vaccine clinic records
Multiple concurrent interventions	• Reduce apparent VE because unvaccinated children are protected • Bias in ecological before-after designs	• Matching by neighbourhood • Recording other concurrent interventions and adjusting in analysis • Adjustment for prevalence of HIV, malaria and malnutrition
High vaccine coverage	• Unvaccinated children differ to general child population	• Commence studies soon after vaccine introduction • Measure and adjust for postulated measurable confounders
Care seeking behaviour and differential ascertainment of disease outcome	• Bias for ecological designs	• Independent verification using alternate data sources of differences in care seeking over time • Propensity scoring
Decline in disease incidence prior to vaccine introduction	• Attribution to vaccine of further declines post vaccine introduction is difficult	• Time series methods • Consistency of results across study designs and study sites • “Dose-response” in decline in incidence with respect to increasing coverage • Extreme caution in causal attribution
Rapid decline in disease following vaccine introduction	• Insufficient case accrual making case-control design unachievable	• Reliance on before-after designs • Utilising secondary endpoints (eg nasopharyngeal carriage rather than invasive disease)
Assuring quality and consistency of data collection at large scale	• May introduce unmeasured bias	• Close and frequent supervision • Well defined management and decision structure • Well defined protocols and operating procedures • Regular review of process measures and key performance indicators
